# Antimicrobial resistance in veterinary medicine: Mechanisms, regulation and control strategies

**DOI:** 10.17221/2/2026-VETMED

**Published:** 2026-06-25

**Authors:** Petra Matlakova, Katerina Nedbalcova

**Affiliations:** ^1^Department of Infectious Diseases and Preventive Medicine, Veterinary Research Institute, Brno, Czech Republic; ^2^Department of Experimental Biology, Faculty of Science, Masaryk University, Brno, Czech Republic

**Keywords:** antibiotics, combination therapy, pharmacodynamics, pharmacokinetics, resistance

## Abstract

Antimicrobial resistance (AMR) represents a major global threat to both human and animal health. Within the One Health framework, farm and wild animals serve as critical reservoirs of resistant bacteria and resistance genes, which can disseminate through food, water, and the environment. The excessive and inappropriate use of antibiotics in veterinary medicine exerts selection pressure that fosters the emergence and spread of antimicrobial resistance. Current mitigation strategies aim to reduce antibiotic consumption and promote prudent use through hygiene measures, vaccination, individualised therapy, pharmacokinetic-pharmacodynamic (PK/PD) dosing optimisation, and comprehensive AMR monitoring. European legislation (EU Regulations 2019/4 and 2019/6) provides stringent guidelines on antibiotic prescription, usage, and residue limits. In this context, antibiotic combination therapy represents a promising approach to enhance antimicrobial efficacy, broaden the antibacterial spectrum, and suppress the emergence of resistance by targeting multiple bacterial pathways simultaneously. In addition to developing new drugs, renewed focus on reviving older antimicrobial molecules*** ***– supported by updated PK/PD data and optimised dosing – could provide an effective alternative in combating multidrug-resistant pathogens.

## INTRODUCTION

The One Health approach is a modern framework linking human health, veterinary medicine, and environmental science. It highlights the close relationship between people, animals, and their shared ecosystems. One of its key priorities is the detection, treatment, and prevention of infectious diseases. Bacterial infections in both humans and animals are typically managed with antibiotics, which remain the primary therapeutic option. However, frequent, excessive, or inappropriate use of antibiotics contributes to the emergence and spread of resistant bacterial populations. These resistant bacteria can subsequently reach humans through the environment or through animal-derived food products, posing risks to public health, livestock production, and food safety (Van Boeckel et al. 2015).

Antibiotic resistance refers to the genetically encoded ability of bacteria to grow in the presence of an antimicrobial agent. In contrast, antibiotic persistence refers to a transient phenotypic state in which a small subpopulation of bacterial cells survives antibiotic exposure without possessing heritable resistance. Persistence may occur when a subpopulation of bacterial cells enters a dormant state because most antimicrobial agents are ineffective against cells that are not actively growing or dividing. Although persistent cells are not genetically resistant, they can contribute to treatment failure and facilitate the development of antimicrobial resistance (Reygaert 2018).

Given the growing concerns surrounding zoonotic diseases, antimicrobial resistance (AMR), and environmental change, it is crucial to acknowledge the role of animals as reservoirs of pathogenic bacteria. Monitoring resistance is an essential component of the One Health approach, which helps to protect both human and animal health in the shared environment (Aslam et al. 2021; Pitt and Gunn 2024).

According to routine reports published by the European Medicines Agency (EMA) and the European Surveillance of Veterinary Antimicrobial Consumption (ESVAC) programme, the use of antibiotics in veterinary medicine across the European Union has steadily declined in recent years. However, differences among EU member states remain. The EMA has set a goal to reduce veterinary antibiotic sales by 50% by 2030. As of 2022, half of this planned reduction has already been achieved, suggesting that the 2030 goal is attainable if the downward trend continues (EMA 2023; EMA 2025a).

The 2023 EMA surveillance report found that 4 380 tonnes of antimicrobial substances were sold for use in animals in the EU, of which 98.4% were intended for livestock. The most frequently sold classes were penicillins (31.4%), tetracyclines (21.6%), and sulphonamides (10.1%). The largest share of antibiotic consumption was recorded in cattle (38.7%), pigs (30.3%), and poultry (18.5%). For comparison, a total of 4 458 tonnes of antibiotics for animal use were sold in 2022. These figures demonstrate an overall decrease in antibiotic sales in the EU. Although consumption remains high, the continuing downward trend represents a positive step forward (EMA 2023; EMA 2025a).

## MECHANISMS OF RESISTANCE AND ECOLOGICAL RELATIONSHIPS

Antibiotic resistance arises from genetic changes in bacterial cells, or through horizontal gene transfer (HGT). Its spread is promoted by the inappropriate and excessive use of antibiotics in medicine, as well as in agriculture. There are two types of antibiotic resistance: primary (natural) resistance, which is typical of a particular type of bacterium, and secondary (acquired) resistance, which arises from mutations or gene transfer between cells (Frieri et al. 2017; MacGowan and Macnaughton 2017; Larsson and Flach 2022).

Antibiotic resistance mechanisms fall into four categories: limitation of drug intake, drug inactivation, modification of the target structure, and active efflux (Rajput et al. 2024). Limitation of drug intake results from the structure of the bacterial cell wall. Gram-negative bacteria have a thick lipopolysaccharide layer on their surface that reduces membrane permeability. In contrast, Gram-positive bacteria possess only a peptidoglycan layer, making them more permeable (Blair et al. 2015).

Drug inactivation can occur through enzyme degradation, whereby enzymes break down the structure of the drug (e.g., beta-lactamases hydrolyse the beta-lactam ring), or through enzyme modification, whereby a chemical group (e.g., acetyl, phosphate, or adenyl) is attached to the antibiotic, rendering it ineffective. This is how *Pseudomonas* spp. and *Enterococcus* spp. can acetylate chloramphenicol (Vivekanandan et al. 2025).

Modification of the target structure involves altering the antibiotic’s binding site, thereby reducing or eliminating its effectiveness. Resistance to beta-lactam antibiotics is often caused by changes in penicillin-binding proteins (PBPs), which reduce drug binding (Gauba and Rahman 2023; Rajput et al. 2024).

Active efflux is the ability of bacteria to transport toxic substances, including antibiotics, out of the cell through transmembrane proteins called efflux pumps. For instance, *Neisseria gonorrhoeae* has the MtrCDE efflux pump, which transports a variety of antibiotics, including penicillins, tetracyclines, third-generation cephalosporins, and azithromycin, out of the cell (Darby et al. 2023).

Bacteria that colonise animals often can form biofilms, which are complex structures of microorganisms that protect themselves from antibiotics and limit their access (Hoiby et al. 2010). In farm and companion animals, biofilms are often associated with chronic infections, including mastitis and respiratory, urinary, and urogenital infections. Biofilms also serve as reservoirs of resistance genes that can spread both between bacterial strains and, subsequently, also between sectors, such as from animals to humans or into the environment (Rychshanova et al. 2022).

Antibiotic resistance genes can be transferred via vertical transfer, from a mother cell to a daughter cell during cell division, or via horizontal transfer. HGT includes, among other things, the process of transformation, whereby bacteria can take up free DNA from the environment (e.g., from water or soil) and incorporate it into their genome. Thus, non-pathogenic bacteria can transfer resistance genes to pathogens, which can then spread further (Punch et al. 2025). The widespread use of antibiotics creates selection pressure in all spheres. Livestock are an important reservoir of resistance genes that can be spread through water, food or direct contact (Cai et al. 2022; Cao et al. 2022; Woolhouse 2024).

Some antibiotics are reserved exclusively for human treatment (e.g., isoniazid for tuberculosis), while others are specific to animals (e.g., ionophores and flavophospholipol) or used in plant production (e.g., streptomycin and tetracyclines). This widespread use across sectors supports the spread of resistance among microorganisms in human, animal, and environmental settings (Aslam et al. 2021).

## STRATEGIES TO LIMIT THE SPREAD OF AMR

One of the most important aspects in the fight against the spread of resistance is ensuring proper hygiene and biosecurity. First, it is necessary to control the entry of people and animals, and to regularly decontaminate or properly process waste to limit the spread of resistant bacteria and their genes. Additionally, efforts should be made to limit the widespread administration of drugs on animal farms and, when possible, favour an individualised approach (Palma et al. 2020; Raasch et al. 2020).

Farm management, including feed and water, is based on voluntarily reducing antibiotic use in animals, ensuring adequate hygiene and breeding density, quarantining infected animals, and taking appropriate measures to prevent disease. Quality feed supports gut microbiota, and administering electrolysed water can help reduce antibiotic consumption (Sucena Afonso et al. 2024).

Vaccination plays an important role in reducing antibiotic consumption by decreasing disease incidence. However, even when vaccines are available for certain diseases, such as respiratory diseases in cattle, antibiotic treatment is often economically advantageous. Currently, most vaccines used on farms target viral infections. Nevertheless, some antibacterial vaccines are also proving essential, particularly against pathogens such as *Mycoplasma hyopneumoniae* in pigs or *Salmonella* spp. and *Pasteurella* spp. in cattle (Kahn et al. 2019).

Lastly, ensuring AMR surveillance and animal health monitoring is important. This means that regular health checks and the early identification of subclinical infections are crucial. Antimicrobial consumption is expected to increase worldwide, particularly among food-producing animals. AMR is a problem without geographical boundaries, so limiting it globally is essential (Van Boeckel et al. 2015; Jacobsen et al. 2023).

Before starting antibiotic treatment, it is necessary to test bacterial susceptibility. A 2013 study found that only 37.8% of veterinarians frequently performed pretreatment susceptibility testing, 44.3% rarely performed testing, and 9.8% never required testing. Some countries do not perform susceptibility testing due to the cost, problems with sample collection, the urgency of the situation, and the unavailability of laboratories or the slow turnaround of laboratory results. Nevertheless, the Federation of Veterinarians of Europe (FVE) urges veterinarians to always perform susceptibility testing before prescribing critically important antibiotics, especially fluoroquinolones and third- and fourth-generation cephalosporins. The European Platform for Responsible Use of Medicines in Animals (EPRUMA) also supports the responsible use of antibiotics and the practice of susceptibility testing (De Briyne et al. 2013).

Before susceptibility testing can begin, samples must be collected correctly and promptly transported to the laboratory. Depending on the type of bacteria, microorganisms are cultivated on specific media (e.g., Mueller–Hinton agar for the disc diffusion method). After about 16–48 h of cultivation, bacteria can be identified. Common susceptibility testing methods include disc diffusion, Etest, microdilution, macrodilution, and agar dilution. In the disc diffusion method, antibiotic-impregnated paper discs are used, and the resulting zone of inhibition is measured. The Etest operates on a similar principle but uses a strip with an antibiotic concentration gradient, allowing determination of the exact antibiotic concentration that inhibits bacterial growth. Microdilution involves serial dilution of the antibiotic in microtiter plates, while macrodilution involves dilution in larger volumes in test tubes. In agar dilution, the antibiotic is applied directly to the agar, and bacterial growth is monitored. The result of dilution sensitivity testing is the MIC (minimum inhibitory concentration), the lowest concentration of an antibiotic required to inhibit bacterial growth. Clinical breakpoints are established values resulting from both diffusion and dilution tests. Diffusion tests classify bacteria into three categories: susceptible (S), intermediate (I), and resistant (R) (Fessler et al. 2023).

In veterinary medicine, antibiotics are used only under precisely defined conditions, such as for treating bacterial infections after a professional examination. Ideally, this examination includes identifying the pathogen and conducting susceptibility tests. Antibiotics can also be used for metaphylactic and prophylactic purposes. Metaphylaxis involves administering treatment to an entire group of animals, following the onset of disease in some of them to prevent the infection from spreading. Prophylaxis is used only in exceptional cases to prevent infection before an outbreak occurs. However, antibiotics must not be used for routine prophylaxis (the preventive administration of antibiotics in the absence of clinical signs of disease is prohibited), to promote animal growth (prohibited in the European Union since 2006), or as a substitute for adequate conditions. Additionally, drugs reserved for human use (e.g., carbapenems, linezolid) must not be administered to animals (Schmerold et al. 2023).

Antimicrobial stewardship (AMS) promotes the responsible and rational use of antibiotics, restricts mass and preventive administration, and prioritises individualised treatment (Lloyd and Page 2018; Caneschi et al. 2023). Some European countries, such as Denmark and the Netherlands, have introduced strict measures, including monitoring antibiotic consumption on farms, setting reduction targets, and prohibiting the use of critically important antibiotic classes in animals. These measures have led to a decrease in antimicrobial resistant bacteria without affecting productivity (Patel et al. 2020). An important aspect of AMS is the application of a pharmacokinetic-pharmacodynamic (PK/PD) approach to optimise dosing and minimise the emergence of resistance (Caneschi et al. 2023).

## OPTIMISATION OF ANTIBIOTIC THERAPY

Pharmacokinetics and pharmacodynamics (PK/PD) are important for optimising the use of antibiotics. Pharmacokinetics describes the fate of the drug in the body – specifically, its absorption, distribution, metabolism, and excretion. Pharmacodynamics, on the other hand, describes the mechanisms of drug action on bacterial cells. Understanding the PK/PD relationship enables us to determine the optimal drug dosage, maximising its effectiveness while minimising the risk of resistance (McKellar et al. 2004).

PK/PD relationships are defined using PK/PD indices, which are specific to individual antibiotic classes. The time above MIC (T > MIC) indicates the percentage of a 24-hour interval during which the antibiotic concentration remains above the MIC. This index is particularly important for beta-lactam antibiotics, whose effect is time-dependent (Nielsen et al. 2011). The ratio of the maximum concentration to the MIC (C_max_/MIC) indicates how many times the maximum antibiotic concentration exceeds the MIC required to suppress bacterial growth. This index is significant for aminoglycosides and fluoroquinolones, for which higher antibiotic concentrations result in a stronger effect. The area under the concentration-to-MIC curve (AUC/MIC) indicates whether the total exposure to the antibiotic exceeds the level required for effective action. This index is important when evaluating the total exposure of a pathogen to a given antibiotic for macrolides, tetracyclines, glycopeptides, and fluoroquinolones (Papich 2014).

Although PK/PD indices are universal, the dose is species-specific. Differences in metabolism, excretion (e.g., via the kidneys or bile), gastrointestinal tract composition (e.g., ruminants vs monogastrics), and physiological parameters may account for differences among animal species. For example, young ruminants eliminate danofloxacin from their bodies approximately three times more slowly than adults. These differences make it impossible to transfer dosage regimens between species or between different age and reproductive categories within a species, without conducting PK studies (Toutain et al. 2021).

PK/PD models are important for designing and optimising dosage regimens for veterinary antimicrobial drugs. They allow for the determination of the exact daily dose, including the administration interval. These models have been successfully applied to several classes of antibiotics, including beta-lactams, fluoroquinolones, amphenicols, tetracyclines, aminoglycosides, macrolides, and colistin. For instance, PK/PD models have been used to determine the precise enrofloxacin dosage for various animal species against different pathogens (Luo et al. 2019).

Furthermore, PK/PD models are used to monitor the excretion of drug metabolites and the presence of their residues in the tissues or products of farm animals. Based on Regulation (EU) 2019/6 of the European Parliament and of the Council on veterinary medicinal products, strict rules have been established regarding withdrawal periods and maximum residue limits (MRLs) for these products. The withdrawal period is the minimum time that must elapse from the last administration of a medicinal product to an animal until food from that animal can be safely obtained. It is important to ensure that drug residue levels in food do not pose a risk to public health. The MRL is the maximum permitted concentration of a drug in food of animal origin [Regulation (EU) 2019/6].

Dosage nomograms are constructed using MIC and PK/PD parameters. These specialised graphical tools are used to determine the appropriate drug dose for a specific animal according to its characteristics, such as weight and age. These nomograms enable rapid, accurate determination of the dose without complex calculations, while accounting for the drug’s PK parameters (clearance, volume of distribution, and target concentration). Thus, treatment effectiveness is optimised, and the risk of toxicity or resistance is minimised (Le Page et al. 2023; Onita et al. 2025).

When setting dosage regimens in veterinary medicine, it is important to consider the recommended PK/PD targets for the main classes of antibiotics. These targets are based on the relationship between PK and PD, and different antibiotic classes use different indicators (Table 1).

**Table 1:** Recommended PK/PD targets for selected classes of antibiotics (Frei et al. 2008; Magreault et al. 2022)

Class of antibiotics	PK/PD index	Recommended target values
Beta-lactams, cephalosporins, monobactams	T > MIC	50% of the dosing interval above MIC
Carbapenems	T > MIC	30% of the dosing interval above MIC
Aminoglycosides	C_max_/MIC	8–10
		
Fluoroquinolones	C_max_/MIC	10–12
	AUC/MIC	125
		
Linezolid	AUC/MIC	100
	T > MIC	85% of the dosing interval above MIC

AUC/MIC = area under the concentration to minimal inhibitory concentration; C_max_/MIC = maximum concentration to the minimal inhibitory concentration; PK/PD = pharmacokinetic-pharmacodynamic; T > MIC = time above minimal inhibitory concentration

## COMBINATION ANTIBIOTIC THERAPY

Combination antibiotic therapy involves using two or more antibiotics to treat a single bacterial infection. Combining these antibiotics produces a synergistic effect, in which their joint action is stronger than that of a single drug (Bonnet and Lourtet-Hascoet 2025). Combination therapy was first introduced in the 1950s to treat tuberculosis with the antibiotics streptomycin and para-aminosalicylic acid and has since become common practice in clinical medicine. In the last five years, up to 30% of all antibiotics approved by the Food and Drug Administration (FDA) were combinations of several active substances (Sullivan et al. 2020).

The main indications for combination therapy include polymicrobial infections, for which a single antibiotic usually does not cover the entire spectrum (e.g., wound infections) (Bonnet and Lourtet-Hascoet 2025). Other important factors include severe, rapidly progressing infections with high mortality and the use of empiric treatment while awaiting pathogen identification and antibiotic susceptibility results. Combination therapy is also used to reduce the risk of developing antibiotic resistance during ongoing treatment (Sullivan et al. 2020). However, excessive and frequent use of combined antibiotic treatment should be limited because it can lead to toxicity, superinfections, selection pressure that promotes resistance, and higher financial costs (Tangden 2014).

When using combination therapy, one must also be aware of antagonism, which is the mutual interaction of drugs that produces opposite effects. For example, using a bacteriostatic antibiotic with a bactericidal antibiotic (e.g., tetracycline and beta- lactam) can cause antagonism. A bacteriostatic tetracycline antibiotic inhibits bacterial growth, thereby reducing the effect of the bactericidal beta-lactam (Ocampo et al. 2014). The administration of fluoroquinolones with substances that cause chelation in the gastrointestinal tract may lead to reduced absorption when administered orally (Pitman et al. 2019). Additionally, coadministration of a macrolide and a lincosamide can reduce effectiveness because both classes of antibiotics bind to the 50S subunit of the ribosome. Thus, when administered simultaneously, there is competition for the ribosome (Tenson et al. 2003).

The most common antibiotic combinations used in combination therapy are trimethoprim and a sulphonamide, a beta-lactam and an aminoglycoside, and a beta-lactam inhibitor and a beta-lactam antibiotic. Trimethoprim-sulphonamide acts by jointly interfering with the synthesis of folic acid in bacterial cells. Since mammalian cells cannot synthesise folic acid, these antibiotics only affect microorganisms (Gustafsson et al. 2024). The advantage of combined beta-lactam and aminoglycoside therapy is that beta-lactam disrupts the bacterial cell wall, facilitating aminoglycoside passage into the cell. A rapid bactericidal effect can be achieved when using a high inoculum. However, the nephrotoxicity and ototoxicity of aminoglycosides remain a problem, so the situation must be monitored (Ishikawa et al. 2024). Beta-lactamase enzymes allow bacteria to quickly develop resistance to beta-lactam antibiotics, which is why these drugs are among the most widely used. Currently, these antibiotics are often used in combination with a beta-lactamase inhibitor. Common combinations include amoxicillin and clavulanic acid, ampicillin and sulbactam, and piperacillin and tazobactam (Bush and Bradford 2019). Tuberculosis treatment is also traditionally based on combination therapy. This treatment is difficult and lengthy. It involves a quadruple combination of isoniazid, rifampicin, pyrazinamide, and ethambutol for two months. This is followed by a double combination of isoniazid and rifampicin for four months (Larkins-Ford and Aldridge 2023).

In veterinary medicine, a combination of penicillin and streptomycin is commonly used to prevent and treat diseases in animals, particularly those intended for food production. This combination is also widely used due to its effectiveness against both Gram-positive and Gram-negative bacteria (Tufa et al. 2023). Trimethoprim and sulphonamide are also used as preoperative prophylaxis in horses. These combinations are further used to treat neonatal sepsis, arthritis, osteomyelitis, diarrhoea, pneumonia, and wound infections. While these combinations are successful in antibiotic therapy, their long-term and frequent use can lead to bacterial resistance (Gustafsson et al. 2024).

Combination therapy is insufficiently described in veterinary medicine in Europe, although it has been described on other continents. A 2023 study from Bangladesh examined bovine brucellosis. Three *Brucella*-positive cows received intrauterine oxytetracycline and simultaneous intramuscular streptomycin and benzylpenicillin treatment. After this combined therapy, the cows tested negative for *Brucella* spp., were successfully inseminated, and gave birth to healthy calves. The authors of the article mention that this therapy is effective, not time-consuming, and could be used on farms (Hussaini et al. 2023). A Chinese study reported similar results when *Streptococcus suis* isolates were obtained from pig carcasses. When testing combined antibiotic therapies, two-drug combinations showed the best synergy. Specifically, one combination involved ampicillin and apramycin, while the other involved tiamulin and spectinomycin. These results demonstrate the potential use of these combinations for treating *S.* *suis* in animals (Yu et al. 2018).

*In vitro* tests are commonly used to evaluate the effects of combination therapy and to determine whether the antibiotics act synergistically, antagonistically, additively, or independently. The FICi (Fractional Inhibitory Concentration index) quantitatively expresses such interactions and is based on the MIC value for each antibiotic, both individually and in combination. The FIC index is calculated as follows: (MIC of drug A in combination/MIC of drug A alone) + (MIC of drug B in combination/MIC of drug B alone). The results are interpreted as follows: FICi ≤ 0.5 indicates synergism, FICi ≥ 4 indicates antagonism, and FICi 0.5–4 indicates no interaction (Drago et al. 2007; Radu et al. 2024).

The time-kill assay and the checkerboard assay are the most common methods used to evaluate the effectiveness of antibiotic combinations. The checkerboard assay is a microtiter plate method that tests combinations of two antibiotics in a grid arrangement. After incubation and determination of bacterial growth, the FIC index is calculated. This method is relatively fast and suitable for screening multiple antibiotic combinations (Bellio et al. 2021). The time-kill assay is a time-dependent killing test that monitors changes in bacterial viability by observing the formation of Colony Forming Units (CFUs)/ml. Samples are taken at different time intervals (e.g., 0, 2, 4, 12, and 24 hours). A decrease in bacterial count of ≥2 log_10_ CFU/ml is usually considered synergism, while an increase of 2 log_10_ CFU/ml is considered antagonism. This method provides a more accurate view of the antibiotic’s effect over time and confirms results from the checkerboard test (Sueke et al. 2010; Brennan-Krohn and Kirby 2019; Kang et al. 2023).

## OLD DRUGS REVISITED

The growing resistance of bacteria to commonly used antibiotics has increased the need to search for new drugs. One possibility is to return to “old antibiotics” – drugs developed and used decades ago that were gradually withdrawn from practice due to side effects, toxicity, or ineffectiveness against resistant bacteria. These substances often have distinct mechanisms of action, enabling targeted use against antibiotic-resistant bacteria, either alone or in combination with newer antibiotics, to achieve a synergistic effect (Boretti and Banik 2025).

Many older antibiotics, developed in the 1950s–1970s gradually fell into oblivion due to the advent of newer, more effective antibiotics with lower toxicity and better tolerance. However, because these drugs have not been evaluated under current regulatory standards, their reuse relies on outdated data, posing a risk to treatment efficacy and safety. Therefore, it is necessary to comprehensively retest these drugs in accordance with current scientific and regulatory requirements before reintroducing them into clinical practice (Theuretzbacher et al. 2015).

Before reintroducing older antibiotics into clinical practice, a suitable, safe drug must be selected that is effective against multidrug-resistant pathogens and has a low risk of resistance development. At the same time, PK/PD data must be updated, dosage must be optimised and clinical studies must be performed for new indications (Zayyad et al. 2017; Trif et al. 2023). Close cooperation with regulatory bodies, such as the EMA and FDA, is also important. This includes reviewing and updating the Summary of Product Characteristics (SmPC). Given that these drugs are often no longer under patent protection, adequate financing of the entire process is also a fundamental requirement (Theuretzbacher et al. 2015; Paranos et al. 2022).

After updating the PK/PD data, it is necessary to optimise the dosage regimen to ensure an effective therapeutic concentration of the antibiotic in the target tissues and blood throughout the entire treatment period. Setting the dosage correctly is important for effective treatment and for minimising the risk of antibiotic resistance. When applying antibiotics to farm animals, the safety of the food chain must also be considered. This means ensuring that antibiotic or metabolite residues in meat, milk, or eggs do not exceed the MRL set by EU legislation, and that the withdrawal period is determined accordingly (Diamantis et al. 2022; Trif et al. 2023).

Currently, several antibiotics are being considered for reintroduction into clinical practice. Colistin is a notable example. First introduced to the market in the 1950s, its use was gradually abandoned due to nephrotoxicity and neurotoxicity. However, the rise of multidrug-resistant bacteria has prompted its re-evaluation. Advances in optimising PK/PD regimens and combination therapies have alleviated previous concerns. Today, colistin is among the key reserve antibiotics used to treat resistant pathogens (Boretti and Banik 2025).

Quinupristin/dalfopristin is a combination of antibiotics that significantly affects Gram-positive bacteria, particularly vancomycin-resistant *Enterococcus faecium.* However, its adverse effects, including vein irritation, arthralgia and myalgia, remain problematic (Bearden 2004; Jovic et al. 2019). Trimethoprim/sulfamethoxazole, developed in the 1960s, is generally of low-toxicity and most commonly causes nausea and vomiting, though hepatotoxicity is also possible (Masters et al. 2003). After adjusting PK/PD parameters and dosage regimens, these antibiotics could be reintroduced into clinical practice, particularly in combinations that mitigate the risk of resistance and adverse effects (Cassir et al. 2014).

If these old drugs are reintroduced into routine medical practice, their use must be carefully monitored. Without monitoring and appropriate dosing regimens, resistance may develop, quickly diminishing their effectiveness. These antibiotics should only be prescribed for severe cases in which treatment of otherwise resistant pathogens is required. In this context, efforts to restore the sensitivity of resistant strains are particularly important (Theuretzbacher et al. 2015).

Antibiotic re-sensitisation aims to restore the sensitivity of resistant bacterial strains to selected antibiotics by lowering their MIC, thus increasing the antibiotics’ effectiveness. This approach is based on knowledge of the resistance mechanisms of specific bacteria and employs various methods to disrupt them, including inhibitors of enzymes that degrade antibiotics, combined therapies, phage therapy, and adjuvants and other chemical substances (Swaminathan et al. 2021). This approach is used in human medicine, for example, when combining a beta-lactam antibiotic with a beta-lactamase inhibitor, and similar principles can also be applied in veterinary medicine (Bognar et al. 2024).

## LEGISLATION AND RECOMMENDATIONS

The European Union’s legislative framework for veterinary medicinal products is currently defined primarily by Regulations (EU) 2019/6 on veterinary medicinal products (European Parliament and Council of the European Union 2019b) and (EU) 2019/4 on medicated feed (European Parliament and Council of the European Union 2019a), which set out rules for their development, registration, use, and supervision. These regulations aim to promote responsible antibiotic use and limit the spread of AMR. Articles 105, 107, and 115 are particularly important within Regulation (EU) 2019/6. Article 105 states that veterinary medicinal products, especially antimicrobial products, may be prescribed only by a veterinarian. The prescription must contain all mandatory information and be limited to the necessary amount. It is also only valid for a limited time to promote the responsible use of medicinal products. Article 107 states that antimicrobial drugs may not be used routinely, even to compensate for deficiencies in hygiene or management practices. Prophylaxis is permitted only in exceptional cases, and metaphylaxis is permitted only when there is a high probability of infection spreading within a group. It also prohibits the use of antimicrobial therapy to promote growth and yield. Article 115 addresses protection periods for veterinary drug use in animal species intended for food production [Regulation (EU) 2019/4; Regulation (EU) 2019/6].

The EMA categorises antibiotics used in animals into four groups (A–D) through the AMEG (Ad Hoc Expert Group), according to the risk of resistance and their importance for public health. Category A (avoid) includes antibiotics prohibited for use in veterinary medicine. Category B (restrict) includes substances that pose a potentially higher risk to the public. Category C (caution) includes antibiotics that can be used when category D antibiotics are unavailable. Category D (prudence) includes antibiotics that should be used as the first choice whenever possible (Table 2) (EMA 2019).

**Table 2:** Categorisation of antibiotics in veterinary medicine according to EMA/AMEG (EMA 2019)

Category	Antibiotics
A – avoid	Beta-lactams: amidinopenicillins, carboxypenicillins and ureidopenicillins (including combinations with beta-lactamase inhibitors), carbapenems, monobactams.
	Other classes: ketolides, rifamycins, lipopeptides, oxazolidinones, riminophenazines, glycopeptides, glycylcyclines, streptogramins, sulfones.
	Specific use: drugs for treating mycobacterial infections, phosphonic acid derivatives.
	
B – restrict	3^rd^- and 4^th^- generation cephalosporins, polymyxins, quinolones and fluoroquinolones.
	
C – caution	Beta-lactams: aminopenicillins with beta-lactamase inhibitors, 1^st^- and 2^nd^- generation cephalosporins.
	Other classes: aminoglycosides (except spectinomycin), amphenicols, lincosamides, pleuromutilins, macrolides, rifaximin.
	
D – prudence	Beta-lactams: aminopenicillins without beta-lactamase inhibitors, narrow-spectrum and anti-staphylococcal penicillins.
	Other classes: tetracyclines, spectinomycin, sulphonamides, cyclic polypeptides, nitroimidazoles, nitrofuran derivatives, fusidic acid.

ESUAvet (European Sales and Use of Antimic-robials for Veterinary Medicine) is a new system that tracks the consumption and use of antimicrobials in animals throughout the EU. It provides an overview of antibiotic use trends across individual member states and evaluates compliance with AMEG recommendations. It also promotes the responsible use of medicines (EMA 2025b).

These regulations directly affect veterinarians in EU Member States. They must adhere to dispensing prescriptions tied to clinical examinations, maintain dispensing documentation, and ensure that prescriptions have limited validity. The administration of medicated feed is subject to special rules regarding duration and the prohibition on its use for prophylaxis. The regulations aim to reduce the use of antimicrobial substances in animals and the spread of AMR without endangering animal health [Regulation (EU) 2019/4 – European Parliament and Council of the European Union 2019a; Regulation (EU) 2019/6 – European Parliament and Council of the European Union 2019b]. However, the new regulations can also lead to various problems. Some medications may not be available in the required formulations, forcing veterinarians to use human medications. Strict prescribing rules, susceptibility testing requirements, and limited options for prophylaxis and metaphylaxis may prevent the rapid implementation of treatment in urgent cases, potentially endangering animal health (More et al. 2022; Nogueira et al. 2024).

## IMPLEMENTATION AND METRICS OF STEWARDSHIP IN PRACTICE

In veterinary AMS, several important quantitative metrics enable monitoring and comparison of antibiotic consumption. The most widely recognised metrics are DDDvet (Defined Daily Dose for animals) and DCDvet (Defined Course Dose for animals). DDDvet allows comparisons between species and countries, whereas DCDvet better reflects real-world practices by defining the dose over the entire treatment cycle (EMA 2016). Another metric is mg/PCU (milligrams per population correction unit), which expresses antibiotic concentration in milligrams (mg) relative to the size of the animal population (EMA 2023).

Used Daily Dose (UDD) and Treatment Incidence (TI) can also be used for a more accurate comparison. UDD indicates the actual doses used in clinical practice, while TI indicates the frequency of treatment in the population (e.g., the number of treated animals per 1 000 days) (Kasabova et al. 2019). Another important AMS indicator is the percentage of targeted therapies based on culture and susceptibility testing. The proportion of critically important antibiotics (CIAs), used to reduce the risk of transferring resistance to human medicine, is also monitored. Tracking consumption by the animal’s type and age allows for targeted interventions (e.g., in weaned pigs or dairy cattle) (EMA 2023).

Standard operating procedures (SOPs) are used to ensure a uniform and responsible approach to these processes. They define the precise steps and conditions that ensure the rational and safe use of antibiotics. SOPs are commonly used in settings that require strict adherence to rules, such as laboratories, production facilities, and research institutions. Examples include procedures for collecting samples, cleaning instruments, preparing samples and sterilising materials. Well-prepared SOPs help maintain the quality and reliability of results and can also serve as training tools for new staff (Hollmann et al. 2020).

## CONCLUSION

AMR remains a critical challenge in veterinary medicine, with profound implications for human and animal health as well as the environment. Although antibiotic use has gradually declined across the European Union, consumption remains high enough to maintain selection pressure and promote the spread of resistant bacteria and genes across different environments and bacterial species. Reliable, region-specific data on antibiotic use and the prevalence of antibiotic resistance are vital for effective AMR management. National and local differences in animal husbandry practices, biosecurity standards, and environmental conditions substantially influence intervention outcomes, underscoring the absence of a universal solution. Despite these concerns, antibiotics remain indispensable in veterinary medicine, as withholding treatment would cause undue animal suffering and compromise welfare. The priority is therefore not merely to reduce use, but to ensure responsible administration – selecting appropriate agents, adjusting dosages, confirming diagnoses, and providing targeted therapy.

Combination antibiotic therapy can reduce selection pressure, limit the emergence of resistance, and expand therapeutic coverage. Given the current paucity of data on combination regimens in animals, further research is warranted to support their integration into veterinary treatment protocols, particularly for severe polymicrobial infections.

Preventive measures must emphasise biosecurity, vaccination, and optimised husbandry, rather than routine prophylactic antibiotic administration. Complementary approaches such as probiotics, phytotherapeutics, and other natural antimicrobial agents warrant further exploration.

Future research should prioritise integrating data from veterinary, human, and environmental sources within the One Health concept. Sustainable AMR control will depend on context-specific, evidence-driven, and regulatory-aligned strategies. Sustainable AMR prevention requires shifting to locally tailored, research- and regulation-informed approaches.
